# A global metabarcoding analysis expands molecular diversity of Platyhelminthes and reveals novel early-branching clades

**DOI:** 10.1098/rsbl.2019.0182

**Published:** 2019-09-11

**Authors:** Konstantina Mitsi, Alicia S. Arroyo, Iñaki Ruiz-Trillo

**Affiliations:** 1 Institut de Biologia Evolutiva (CSIC-Universitat Pompeu Fabra), Passeig Marítim de la Barceloneta 37-49, Barcelona 08003, Catalonia, Spain; 2 Departament de Genètica, Microbiologia i Estadística, Facultat de Biologia, Institut de Recerca de la Biodiversitat (IRBio), Universitat de Barcelona (UB), Barcelona 08028, Catalonia, Spain; 3 ICREA, Pg. Lluís Companys 23, Barcelona 08010, Catalonia, Spain

**Keywords:** flatworms, barcoding, 18S rDNA, marine, freshwater, phylogenetic placement

## Abstract

Understanding biological diversity is crucial for ecological and evolutionary studies. Even though a great part of animal diversity has already been documented, both morphological surveys and metabarcoding analyses have previously shown that some animal groups, such as Platyhelminthes, may harbour hidden diversity. To better understand the molecular diversity of Platyhelminthes, one of the most diverse and biomedically important animal phyla, we here combined data from six marine and two freshwater metabarcoding expeditions that cover a broad variety of aquatic habitats and analysed the data by phylogenetic placement. Our results show that a great part of the hidden diversity is located in early-branching clades such as Catenulida and Macrostomorpha, as well as in late-diverging clades such as Proseriata and Rhabdocoela. We also report the first freshwater record of Gnosonesimida, a group previously thought to be exclusively marine. Finally, we identified two putative novel freshwater Platyhelminthes clades that branch between well-defined orders of the phylum. Thus, our analyses of several environmental datasets confirm that a large part of the diversity of Platyhelminthes remains undiscovered, point to groups with more potential novel species and identify freshwater environments as potential reservoirs for novel species of flatworms.

## 1. Introduction

To understand past and present biological processes and to make meaningful decisions for the future, it is of pivotal importance to decipher extant biodiversity [1]. Accurate biodiversity assessment is difficult because of sampling biases and the limitations of morphology-based taxonomic identification [2]. Sampling biases include restrictions in sampling site accessibility and preferential collection of specimens due to methodological constraints, both of which lead to a non-representative sample of the community under study. Traditional identification methods based on morphology are low throughput, time and resource consuming, require high taxonomic expertise that is particularly rare for most of the groups that are not well studied and fail to cope with cryptic diversity. As a consequence, it is estimated that real extant species diversity probably exceeds by 10-fold the current number of described species [3]. Although the unicellular eukaryotic lineages suffer from this bias more than their multicellular counterparts [4,5], there are groups of animals for which an assessment of diversity is incomplete [6,7].

Platyhelminthes (flatworms) is one of the most diverse [8] and relatively well-studied animal phyla. Initially considered to be an early-branching bilaterian clade because of their simple morphology, they were studied to understand the origin of bilaterian symmetry. However, recent molecular phylogenies nested them inside the superphylum Lophotrochozoa (Spiralia) [9–11] and recognized them as secondarily simplified. In addition, species of flatworms are considered as model organisms to study whole-body regeneration [12,13] and the evolution of development [14]. Flatworms are also biomedically relevant, being 75% of the described species of obligate parasites of vertebrates [15,16]. Ecologically, they are key meiofaunal taxa in both marine and freshwater aquatic environments [17,18]. Nevertheless, flatworms have rarely been taken into consideration in traditional biodiversity studies, given that their morphological identification is tedious, requiring fixation and histological processing [19] or live examination when fixation can destroy their taxonomically informative internal reproductive anatomy [18]. Current estimates for the species richness of this phylum suggest that there is quite a lot of hidden diversity yet to be identified [7].

Metabarcoding emerged as a promising solution to unravelling hidden diversity and has been successfully applied in different groups of organisms and habitats [20–24]. However, to our knowledge, a thorough analysis of metabarcoding data on Platyhelminthes has never been done. To fill this gap, we here analysed different environmental datasets from six marine and two freshwater habitats to both explore the diversity of Platyhelminthes at the level of orders and detect potential novel, undescribed molecular diversity.

## 2. Material and methods

We collected a total of 1380 representative sequences of clustered operational taxonomic units (OTUs) identified as Platyhelminthes in six marine and two freshwater environmental surveys. Each survey targeted a different hypervariable region of the 18S rRNA gene (table 1, [25–31]). We assigned sequences to either groups or taxonomic categories based on BLAST 2.6.0 [32] searches of the SILVA 128 SSU reference database [33,34].

**Table 1.** Sampling and filtering information.

dataset	habitat	18S rRNA variable region	no. OTUs before filtering	no. OTUs after filtering	reference
TaraOceans	global marine water column	V9	185	117	de Vargas *et al*. [25]
BioMarks	European coastal benthos and marine water column	V4	33	33	Massana *et al*. [26]
Metabarpark	﻿Atlantic and Mediterranean marine hard-bottom benthos	V7	232	1	Wangensteen *et al*. [27]
DOSMARES	Mediterranean marine deep sea benthos (Blanes canyon)	V7	123	1	Guardiola *et al*. [28]
INDEMARES	Mediterranean marine deep sea benthos	V7	29	0	Guardiola *et al*. [29]
DeepSea	Atlantic and Pacific marine deep sea benthos	V8–V9	80	17	Bik *et al*. [30]
Parana	river in Argentina	V4	14	7	Arroyo *et al*. [31]
Sanabria	glaciar lake in Iberian peninsula	V4	684	667	in preparation

We constructed a reference tree of complete 18S rDNA sequences retrieved from the National Center for Biotechnology Information (NCBI) nucleotide database to use as a backbone for phylogenetic placement. We aimed to include all the known extant diversity of Platyhelminthes focusing on the free-living representatives. To this end, we conducted a bibliographic search [35–37] to select complete sequences of all the known families for each order of the phylum. We restricted our taxon sampling to 455 taxa so that the resulting reference tree could easily be visually inspected, while encompassing the diversity of the extant Platyhelminthes according to the latest complete phylogeny of the phylum [16,38].

We filtered our initial metabarcoding dataset both by alignment (using PaPaRa v. 2.5 [39] to align the query sequences to the reference alignments) and by phylogenetic placement (using the EPA [40] as implemented in RAxML [41]). All trimming was done with trimAL [42]. We removed all sequences that (i) did not align in the correct hypervariable region, (ii) were placed in the outgroup of the reference tree, (iii) had extremely long branches in the best-hit placement tree making the rate of nucleotide substitutions greater than 1, and (iv) had a placement hit both in Platyhelminthes branches but also in the outgroup branches. The filtered dataset (843 OTUs) was placed onto the reference tree using two different phylogenetic placement algorithms, RAxML-EPA [40] and pplacer [43]. We compared the resulting jplace files using compare_jplace_files.cpp as implemented in genesis tool (http://genesis-lib.org/) to confirm that the placement with the highest likelihood–weight ratio (top placement) of both Pqueries was located on the same branch.

We constructed maximum-likelihood trees for all the queries with top placement outside the known Platyhelminthes orders: (i) a tree in which we combined full-length reference sequences with short queries and (ii) a tree in which we manually trimmed the reference alignment to the length of the short queries. The trees were built (i) in RAxML [41] under the GTR + GAMMA substitution model with 1000 rapid bootstrap replicates and (ii) in IQTREE [44] under the TN + F+R8 substitution model with 1000 ultrafast bootstraps and tested tree branches by SH-like aLRT with 1000 replicates. All trees were visualized in iTOL [45].

## 3. Results and discussion

We used 18S rDNA metabarcoding data from aquatic environments to expand our understanding of the molecular diversity of Platyhelminthes, search for novel lineages and identify potential diversity hotspots for future expeditions. To this end, we compiled the most comprehensive flatworm metabarcoding 18S rDNA dataset to date, comprising 1380 query sequences from six marine and two freshwater environmental surveys (table 1).

**Figure 1.**
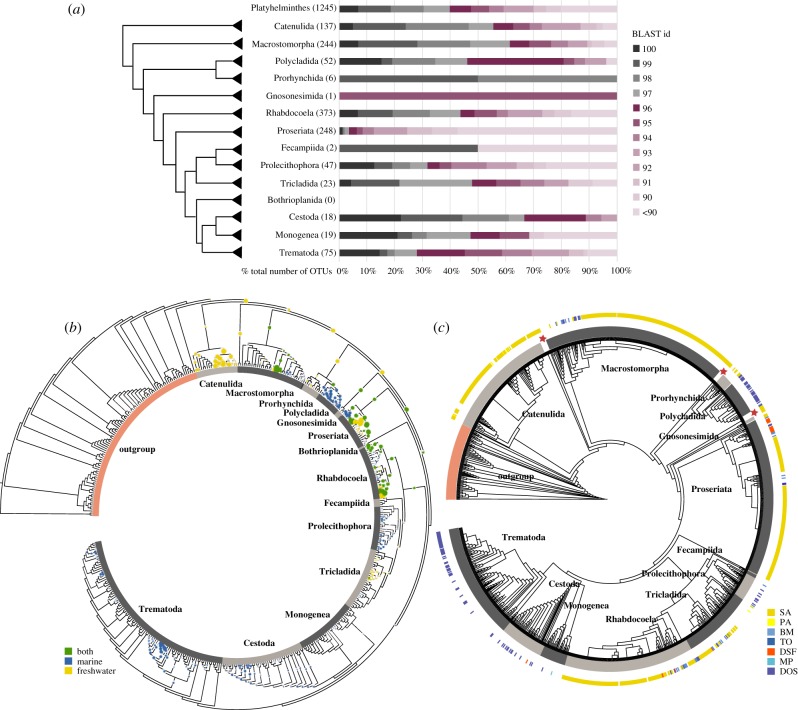
Molecular diversity and novelty in Platyhelminthes. (*a*) BLAST novelty. Stacked barplots represent the distribution of nucleotide BLAST identity percentages of the queries. The total number of sequences for every group is shown in parentheses. Note that 60% of the total OTUs identified as Platyhelminthes had less than 97% identity to the reference database. (*b*) Phylogenetic placement novelty. A total of 843 query sequences were placed into the reference tree. The colour code of the placements reflects the habitat of origin. Placements expand the molecular diversity of Polycladida, Proseriata and Rhabdocoela and indicate novelty in the internal nodes of early-branching clades. (*c*) Best-hit placement tree. This placement tree is based on the highest likelihood–weight ratio for each query. The inner circle (in grey) reflects the limits of Platyhelminthes orders. The outer coloured circle shows the leaves of the tree that correspond to query sequences. The colour code stands for the dataset of origin for each query: SA, Sanabria; PA, Parana; BM, Biomarks; TO, TaraOcean; DSF, DeepSea; MP, Metabarpark; DOS, DOSMARES. All queries marked with a star do not correspond to any known order. (Online version in colour.)

We first checked how many of those sequences corresponded to taxa already described and sequenced and how many represented novel taxa. We performed BLASTn searches against the SILVA 128 SSU database to confirm the identity of these potential Platyhelminthes. Among the 1245 query sequences that returned a flatworm sequence as the first hit, 60% had less than 97% sequence identity with the reference sequences (figure 1*a*). All groups, both parasitic and free living, showed high percentages of sequences with low BLAST identity (less than 97% sequence identity), Proseriata, Prolecithophora and Trematoda being the groups with the highest percentages. For example, 95% of the 248 Proseriata sequences had less than 97% sequence identity with reference sequences.

The quality of the reference database is of pivotal importance to evaluate the number of novel species inside a clade. In biodiversity assessments based on sequence similarity methods, a good reference database includes as many sequences as possible. By contrast, to evaluate the number of novel species using a phylogeny-driven approach, the number of taxa in a good reference tree should be small enough to allow the visual inspection of the results and broad enough to encompass all existing diversity. To this end, we inferred our Platyhelminthes 18S rDNA reference tree based on a broad taxon sampling of 455 complete 18S rDNA sequences including representatives from all major flatworm clades. Although our reference tree did not recover the same topology of orders as in multigene phylogenetic analyses [16,38], most orders were monophyletic. This is important for the subsequent placement analysis as all the queries that fall into the known delimited orders expand the diversity inside these orders and all the queries that fall between well-defined orders represent completely novel molecular diversity.

Given that each study targeted different hypervariable regions of the 18S rRNA gene, our initial dataset was a mixture of V4, V7, V8–V9 and V9 18S rDNA queries (table 1). Queries from a hypervariable region must map to a full-length 18S rDNA with minimal ambiguity to serve as a reliable phylogenetic marker. However, in many cases, queries do not align unambiguously because of the fast-evolving nucleotide sites resulting in unreliable trees. To overcome this pitfall, we refined the unfiltered dataset of 1245 query sequences by alignment. More than one-quarter of the initial sequences were removed because of misalignment (table 1). The majority of V7 queries were removed during this filtering step, indicating that the V7 hypervariable region was too short and variable to be useful as a molecular marker for Platyhelminthes. By contrast, all V4 and V9 queries were retained, showing that these variable regions can serve as quality molecular markers for Platyhelminthes.

Our phylogenetic placement analyses showed that the majority of OTUs grouped with free-living taxa (figure 1*b*). We detected phylogenetic placements in the internal nodes of early-branching clades such as Catenulida, Macrostomorpha, Prorhynchida and Polycladida that potentially indicate novel groups yet to be described. Many OTUs grouped within Polycladida, a well-described clade with more than 800 described species, of which only 30 have representative sequences. Thus, these placements probably reflect a lack of molecular data in the reference database rather than real novel diversity. Many other OTUs were placed within Proseriata and Rhabdocoela, probably representing novel diversity, given that these two clades are well sampled for the 18S rRNA gene.

We then inquired whether we could detect marine OTUs in groups considered freshwater and vice versa (figure 1*b,c*). As expected, marine groups were only detected in marine samples and freshwater groups in freshwater samples, except for one clade, Gnosonesimida. We recovered the first freshwater record of Gnosonesimida, a group formed by only six described species thought to be exclusively marine [16,18]. Polycladida and Prolecithophora have both freshwater and marine representatives, but we could only detect them in our marine datasets. Even though Neodermata is formed by obligate parasites, we recovered OTUs in the marine water column. Those OTUs were placed inside the three orders of Neodermata, suggesting that free-living stages may have been sampled.

**Figure 2.**
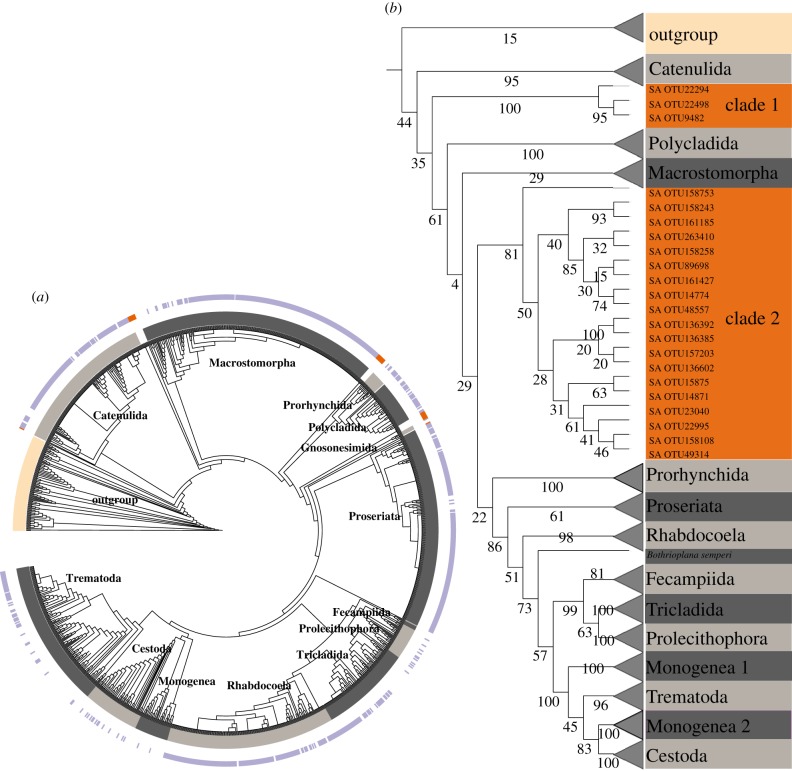
Two novel freshwater clades. (*a*) OTUs with interesting placements. The inner circle (in grey) reflects the limits of Platyhelminthes orders. In the outer circle, purple indicates the leaves that correspond to OTUs and no colour the leaves that correspond to reference sequences. The orange marks highlight the OTUs that were placed outside the known flatworm orders in the best-hit placement tree. (*b*) Maximum-likelihood tree. The tree was inferred from 22 OTUs with interesting placements and 455 reference sequences. Nodal support indicates 1000 maximum-likelihood rapid bootstrap replicates. Orange clades represent novel molecular lineages within Platyhelminthes. (Online version in colour.)

We then analysed potential novel clades within Platyhelminthes. In our best-hit placement tree (figure 1*c*), we localized OTUs that were placed outside the limits of known flatworm orders (figure 2*a*) with high likelihood–weight scores and characterized them as ‘interesting placements'. We inferred a maximum-likelihood tree from the alignment of the full-length reference sequences and those OTUs with phylogenetically interesting placements (figure 2*b*). The phylogenetic tree revealed two novel freshwater clades, clade 1 and clade 2, branching as monophyletic clades in between major Platyhelminthes orders. Clade 1 was formed by three sequences that had 92% sequence identity with sequences of the genus Castrada (Typhloplanidae) and clade 2 by 19 sequences with sequence identity to *Otomesostoma auditivum* and *Invenusta aestus* (Coelogynoporidae) that ranged between 91% and 94%. We also inferred a maximum-likelihood tree using only the V4 hypervariable region of the reference alignment and the short queries; this tree was not informative because of the weak phylogenetic signal. Even though the exact phylogenetic position of the new clades within Platyhelminthes remains unclear, they certainly form two separate, well-defined groups, probably in early-branching positions.

Phylogenetic placement outperforms the conceptually problematic but often used practice of reconstructing de novo phylogenies from short reads that do not contain sufficient phylogenetic signal to reproduce a reasonable tree. It is a reliable method to classify short DNA sequences, a common output of metabarcoding and metagenomic studies, and has been extensively used for taxonomic assignment in diversity studies. Overall, our analyses show a high diversity of Platyhelminthes in both marine and freshwater environments, with the latter habitat likely containing as yet unnamed taxa. We found that Proseriata and Rhabdocoela are the two flatworm groups with more potential novel species. Our data also show a high novelty of molecular data in Catenulida and Macrostomorpha that may correspond either to unsequenced data or to new taxa. Moreover, we identified, in freshwater environments, two novel clades that group outside the well-known Platyhelminthes orders. While our data demonstrate the utility of metabarcoding analyses in search of novel diversity, we emphasize the need for more traditional taxonomic efforts to have a good understanding of animal diversity.

## Supplementary Material

Placement files and comparison

## Supplementary Material

Placement files and comparison

## Supplementary Material

Reference tree alignment

## Supplementary Material

PaPaRa alignment of short reads

## Supplementary Material

Placement files and comparison

## Supplementary Material

Maximum-likelihood tree with new clades

## Supplementary Material

Best-hit placement tree

## Supplementary Material

Best-hit placement tree

## Data accessibility

Alignments, trees and jplace files are available as electronic supplementary material.

## References

[1] Pawlowski J, Lejzerowicz F, Apotheloz-Perret-Gentil L, Visco J, Esling P 2016 Protist metabarcoding and environmental biomonitoring: time for change. Eur. J. Protistol. 55, 12–25. (10.1016/j.ejop.2016.02.003) 27004417

[2] Wangensteen OS, Turon X 2016 Metabarcoding techniques for assessing biodiversity of marine animal forests. In Marine animal forests (eds Rossi S, Bramanti L, Gori A, Orejas C del Valle Saco), pp. 1–29. Cham, Switzerland: Springer International Publishing.

[3] Appeltans W et al. 2012 The magnitude of global marine species diversity. Curr. Biol. 22, 2189–2202. (10.1016/J.CUB.2012.09.036) 23159596

[4] Pawlowski J et al. 2012 CBOL Protist Working Group: barcoding eukaryotic richness beyond the animal, plant, and fungal kingdoms. PLoS Biol. 10, e1001419 (10.1371/journal.pbio.1001419) 23139639 PMC3491025

[5] Del Campo J, Sieracki ME, Molestina R, Keeling P, Massana R, Ruiz-Trillo I 2014 The others: our biased perspective of eukaryotic genomes. Trends Ecol. Evol. 29, 252–259. (10.1016/j.tree.2014.03.006) 24726347 PMC4342545

[6] Arroyo AS, López-Escardó D, de Vargas C, Ruiz-Trillo I 2016 Hidden diversity of Acoelomorpha revealed through metabarcoding. Biol. Lett. 12, 20160674 (10.1098/rsbl.2016.0674) 27677819 PMC5046940

[7] López-Escardó D, Paps J, de Vargas C, Massana R, Ruiz-Trillo I, del Campo J 2018 Metabarcoding analysis on European coastal samples reveals new molecular metazoan diversity. Sci. Rep. 8, 9106 (10.1038/s41598-018-27509-8) 29904074 PMC6002407

[8] Zhang Z-Q 2011 Animal biodiversity: an introduction to higher-level classification and taxonomic richness. Zootaxa 3148, 7–12. (10.11646/zootaxa.3148.1.3) 26146682

[9] Carranza S, Baguñi J, Riutort M 1997 Are the Platyhelminthes a monophyletic primitive group? An assessment using 18s rDNA sequences. Mol. Biol. Evol. 14, 485–497. (10.1093/oxfordjournals.molbev.a025785) 9159926

[10] Ruiz-Trillo I, Riutort M, Littlewood DT, Herniou EA, Baguña J 1999 Acoel flatworms: earliest extant bilaterian Metazoans, not members of Platyhelminthes. Science 283, 1919–1923. (10.1126/science.283.5409.1919) 10082465

[11] Dunn CW et al. 2008 Broad phylogenomic sampling improves resolution of the animal tree of life. Nature 452, 745–749. (10.1038/nature06614) 18322464

[12] Sánchez Alvarado A 2012 What is regeneration, and why look to planarians for answers? BMC Biol. 10, 88 (10.1186/1741-7007-10-88) 23136835 PMC3493261

[13] Saló E, Baguñà J 2002 Regeneration in planarians and other worms: new findings, new tools, and new perspectives. J. Exp. Zool. 292, 528–539. (10.1002/jez.90001) 12115936

[14] Martín-Durán JM, Egger B 2012 Developmental diversity in free-living flatworms. Evodevo 3, 7 (10.1186/2041-9139-3-7) 22429930 PMC3379954

[15] Littlewood DTJ, Rohde K, Bray RA, Herniou EA 1999 Phylogeny of the Platyhelminthes and the evolution of parasitism. Biol. J. Linn. Soc. 68, 257–287. (10.1111/j.1095-8312.1999.tb01169.x)

[16] Laumer CE et al. 2015 Nuclear genomic signals of the ‘microturbellarian’ roots of platyhelminth evolutionary innovation. Elife 4, 2189–2202. (10.7554/eLife.05503) PMC4398949 25764302

[17] Fonseca VG et al. 2010 Second-generation environmental sequencing unmasks marine metazoan biodiversity. Nat. Commun. 1, 98 (10.1038/ncomms1095) 20981026 PMC2963828

[18] Schockaert ER, Hooge M, Sluys R, Schilling S, Tyler S, Artois T 2008 Global diversity of free living flatworms (Platyhelminthes, ‘Turbellaria’) in freshwater. Hydrobiologia 595, 41–48. (10.1007/s10750-007-9002-8)

[19] Scarpa F, Cossu P, Delogu V, Lai T, Sanna D, Leasi F, Norenburg JL, Curini-Galletti M, Casu M 2017 Molecular support for morphology-based family-rank taxa: the contrasting cases of two families of Proseriata (Platyhelminthes). Zool. Scr. 46, 753–766. (10.1111/zsc.12251)

[20] Mahé F et al. 2017 Parasites dominate hyperdiverse soil protist communities in Neotropical rainforests. Nat. Ecol. Evol. 1, 0091 (10.1038/s41559-017-0091) 28812652

[21] Pornon A et al. 2016 Using metabarcoding to reveal and quantify plant–pollinator interactions. Sci. Rep. 6, 27282 (10.1038/srep27282) 27255732 PMC4891682

[22] Beng KC, Tomlinson KW, Shen XH, Surget-Groba Y, Hughes AC, Corlett RT, Slik JWF 2016 The utility of DNA metabarcoding for studying the response of arthropod diversity and composition to land-use change in the tropics. Sci. Rep. 6, 24965 (10.1038/srep24965) 27112993 PMC4844954

[23] Bucklin A, Lindeque PK, Rodriguez-Ezpeleta N, Albaina A, Lehtiniemi M 2016 Metabarcoding of marine zooplankton: prospects, progress and pitfalls. J. Plankton Res. 38, 393–400. (10.1093/plankt/fbw023)

[24] Shaw JLA, Clarke LJ, Wedderburn SD, Barnes TC, Weyrich LS, Cooper A 2016 Comparison of environmental DNA metabarcoding and conventional fish survey methods in a river system. Biol. Conserv. 197, 131–138. (10.1016/j.biocon.2016.03.010)

[25] de Vargas C et al. 2015 Eukaryotic plankton diversity in the sunlit ocean. Science 348, 1261605 (10.1126/science.1261605) 25999516

[26] Massana R et al. 2015 Marine protist diversity in European coastal waters and sediments as revealed by high-throughput sequencing. Environ. Microbiol. 17, 4035–4049. (10.1111/1462-2920.12955) 26119494

[27] Wangensteen OS, Palacín C, Guardiola M, Turon X 2018 DNA metabarcoding of littoral hard-bottom communities: high diversity and database gaps revealed by two molecular markers. PeerJ 6, e4705 (10.7717/peerj.4705) 29740514 PMC5937484

[28] Guardiola M, Wangensteen OS, Taberlet P, Coissac E, Uriz MJ, Turon X 2016 Spatio-temporal monitoring of deep-sea communities using metabarcoding of sediment DNA and RNA. PeerJ 4, e2807 (10.7717/peerj.2807) 28028473 PMC5180584

[29] Guardiola M, Uriz MJ, Taberlet P, Coissac E, Wangensteen S, Deep-Sea TX 2015 Deep-sequencing: metabarcoding extracellular DNA from sediments of marine canyons. PLoS ONE 10, e0139633 (10.5061/dryad.520gq) 26436773 PMC4593591

[30] Bik HM, Sung WA, De Ley P, Baldwin JG, Sharma J, Rocha-Olivares AX, Thomas WK 2012 Metagenetic community analysis of microbial eukaryotes illuminates biogeographic patterns in deep-sea and shallow water sediments. Mol. Ecol. 21, 1048–1059. (10.1111/j.1365-294X.2011.05297.x) 21985648 PMC3261328

[31] Arroyo AS, López-Escardó D, Kim E, Ruiz-Trillo I, Najle SR 2018 Novel diversity of deeply branching Holomycota and unicellular holozoans revealed by metabarcoding in Middle Paraná River, Argentina. Front. Ecol. Evol. 6, 99 (10.3389/fevo.2018.00099)

[32] Koski LB, Golding GB 2001 The closest BLAST hit is often not the nearest neighbor. J. Mol. Evol. 52, 540–542. (10.1007/s002390010184) 11443357

[33] Quast C, Pruesse E, Yilmaz P, Gerken J, Schweer T, Yarza P, Peplies J, Glöckner FO 2012 The SILVA ribosomal RNA gene database project: improved data processing and web-based tools. Nucleic Acids Res. 41, D590–D596. (10.1093/nar/gks1219) 23193283 PMC3531112

[34] Yilmaz P et al. 2014 The SILVA and ‘All-species Living Tree Project (LTP)’ taxonomic frameworks. Nucleic Acids Res. 42, D643–D648. (10.1093/nar/gkt1209) 24293649 PMC3965112

[35] Littlewood DTJ 2008 Platyhelminth systematics and the emergence of new characters. Parasite 15, 333–341. (10.1051/parasite/2008153333) 18814704

[36] Larsson K, Jondelius U 2008 Phylogeny of Catenulida and support for Platyhelminthes. Org. Divers. Evol. 8, 378–387. (10.1016/j.ode.2008.09.002)

[37] Janssen T, Vizoso DB, Schulte G, Littlewood DT, Waeschenbach A, Schärer L 2015 The first multi-gene phylogeny of the Macrostomorpha sheds light on the evolution of sexual and asexual reproduction in basal Platyhelminthes. Mol. Phylogenet. Evol. 1, 82–107. (10.1016/j.ympev.2015.06.004) 26093054

[38] Egger B et al. 2015 A transcriptomic-phylogenomic analysis of the evolutionary relationships of flatworms. Curr. Biol. 25, 1347–1353. (10.1016/j.cub.2015.03.034) 25866392 PMC4446793

[39] Berger SA, Stamatakis A 2011 PaPaRa 2.0: a vectorized algorithm for probabilistic phylogeny-aware alignment extension. http://sco.h-its.org/exelixis/pubs/Exelixis-RRDR-2012-5.pdf.

[40] Berger SA, Krompass D, Stamatakis A 2011 Performance, accuracy, and Web Server for evolutionary placement of short sequence reads under maximum likelihood. Syst. Biol. 60, 291–302. (10.1093/sysbio/syr010) 21436105 PMC3078422

[41] Stamatakis A 2014 RAxML version 8: a tool for phylogenetic analysis and post-analysis of large phylogenies. Bioinformatics 30, 1312–1313. (10.1093/bioinformatics/btu033) 24451623 PMC3998144

[42] Capella-Gutiérrez S, Silla-Martínez JM, Gabaldón T 2009 trimAl: a tool for automated alignment trimming in large-scale phylogenetic analyses. Bioinformatics 25, 1972–1973. (10.1093/bioinformatics/btp348) 19505945 PMC2712344

[43] Matsen FA, Kodner RB, Armbrust V 2010 pplacer: linear time maximum-likelihood and Bayesian phylogenetic placement of sequences onto a fixed reference tree. BMC Bioinf. 11, 538 (10.1186/1471-2105-11-538) PMC3098090 21034504

[44] Nguyen L-T, Schmidt HA, von Haeseler A, Minh BQ 2015 IQ-TREE: a fast and effective stochastic algorithm for estimating maximum-likelihood phylogenies. Mol. Biol. Evol. 32, 268–274. (10.1093/molbev/msu300) 25371430 PMC4271533

[45] Letunic I, Bork P 2016 Interactive tree of life (iTOL) v3: an online tool for the display and annotation of phylogenetic and other trees. Nucleic Acids Res. 44, W242–W245. (10.1093/nar/gkw290) 27095192 PMC4987883

